# Efficacy and safety of baricitinib in treatment of systemic lupus erythematosus: a systematic review and meta-analysis

**DOI:** 10.1186/s41927-023-00363-6

**Published:** 2023-10-31

**Authors:** Abdallah R. Allam, Mohamed Salah Alhateem, Abdelrahman Mohamed Mahmoud

**Affiliations:** Faculty of Medicine, Menoufia University, Yassin Abdelghaffar Street from Gamal Abdelnaser Street. Shebin Al- Kom, Menoufia, 32511 Egypt

**Keywords:** Baricitinib, Systemic Lupus Erythematosus, SLE, SRI-4, Systematic review, Meta-analysis

## Abstract

**Background:**

SLE is an autoimmune disease marked by broad immunological dysregulation and multi-system inflammation. Baricitinib is one of the novel treatments for SLE. We conducted this meta-analysis to evaluate its safety and effectiveness in treating SLE.

**Method:**

We looked for all published randomized controlled trials in PubMed, Scopus, Web of Science, and Cochrane and included all RCTs comparing baricitinib and placebo in the treatment of SLE. Review Manager 5.4 program was used for data analysis.

**Results:**

Three trials with a total of 1849 individuals were included. Participants in the baricitinib group were significantly more likely to attain SRI-4 response than those in the placebo group [RR = 1.11, 95% CI (1.02, 1.21), P = 0.01]. Additionally, baricitinib performed better than the placebo in terms of reduction of ≥ 4 points from baseline in SLEDAI-2 K score [RR = 1.13, 95% CI (1.04, 1.22), P = 0.004]. In terms of SLEDAI-2 K remission of arthritis or rash, baricitinib was also superior to placebo [RR = 1.08, 95% CI (1.00, 1.17), P = 0.04]. Treatment-emergent adverse events did not differ significantly [RR = 1.01, 95% CI (0.97, 1.05), P = 0.61].

**Conclusion:**

Baricitinib is potentially safe and effective in the treatment of SLE. It has successfully met the study’s primary endpoint and some secondary endpoints highlighting its potential to improve the outcomes of SLE. Despite achieving an SRI-4 response, glucocorticoids sparing and some other secondary outcomes weren’t reached by baricitinib.

**Supplementary Information:**

The online version contains supplementary material available at 10.1186/s41927-023-00363-6.

## Introduction

Systemic lupus erythematosus (SLE) is a chronic autoimmune disease that affects numerous body organs and systems [1]. It is caused by the interplay between genetic, immunological, and environmental factors and presents with a wide range of symptoms like joint pain, skin rash, and multi-organ damage [2].

SLE has no known cause or treatment; nevertheless, there are several ways to manage the condition and slow its progression. These drugs include a combination of NSAIDs, anti-malarial drugs, corticosteroids, and immunosuppressive agents [3]. However, these medications are linked to substantial adverse events, and many patients suffer from disease relapse and symptom flare-ups [4].

The development of new treatments for SLE has been challenging, with few advancements in the past six decades. Only two new treatments approved in the last 60 years targeting B cells and type 1 interferon highlighting the unmet need for the development of new SLE therapies [5, 6].

The pathogenesis of SLE is complex, involving various immune mechanisms and factors underlying the disease activity. Different patients and clinical presentations exhibit variations in the dominance of these mechanisms. Consequently, the existing limited range of therapeutic targets does not cater to all patients. Extensive research has implicated a range of cytokines, including interferons, B-cell activating factors, various interleukins, and TNF in the development of SLE [7–9]. Many of these cytokines rely on Janus kinases (JAKs) for intracellular signaling [1, 10, 11].

Janus kinase inhibitors, a group of drugs approved by the US Food and Drug Administration, have shown an effective role in treating many inflammatory and autoimmune diseases, such as rheumatoid arthritis and psoriasis [3, 12]. They work by blocking the pathways of several cytokines, including interleukin (IL)-6 and interferon and interferon (IFN)-γ, which are involved in the pathogenesis of SLE [11, 13].

Baricitinib, one of Janus kinase inhibitors, has emerged as a potential therapeutic option for the treatment of a wide scale of autoimmune conditions including SLE [14–17]. A number of clinical trials have been carried out to assess the safety and efficacy of baricitinib in the treatment of SLE [15–17]. However, the results of these trials were conflicting, Morand et al. reported potential effectiveness of baricitinib 4 mg in treatment of SLE which was supported by Wallace et al. On the other hand, Petri et al. reported failure of baricitinib to meet the primary end point of the study. Therefore, it is still uncertain if baricitinib is effective for SLE treatment.

We carried out this comprehensive systematic review to compare the safety and effectiveness of baricitinib versus placebo in the treatment of SLE. Moreover, we conducted a meta-analysis of available data to estimate the effect of baricitinib on disease activity, flare-ups, and adverse events.

## Methods and materials

We performed this systematic review according to Cochrane guidelines [18] and preferred reporting items for systematic reviews and meta-analyses (PRISMA) guidelines [19].

### Literature search and screening

We performed a systematic search on PubMed, Scopus, Web of Science, and Cochrane till March 2023. Entry terms were as follows: (“Systemic Lupus Erythematosus,” “Lupus Erythematosus Disseminatus,” “Libman-Sacks Disease,” “Disease, Libman-Sacks,” “Libman Sacks Disease,” “baricitinib,” “Olumiant,” “baricitinib phosphate,” “baricitinib phosphate salt.”

In order to find pertinent publications for our systematic review, we carried out a four-step screening procedure. In the initial phase, we collected all retrieved articles and eliminated duplicates using Endnote 20 software. Next, the articles’ titles and abstracts were inspected to rule out studies that weren’t relevant. In the third step, we examined the complete texts of the remaining articles to determine whether they qualified for the review. Finally, we looked over the reference lists of the articles that were included to identify any potentially relevant publications that were missed in the initial search. The screening process was conducted independently by two researchers using the SR Accelerator tool, and any disagreements were resolved by a third researcher [20].

### Eligibility criteria

We restricted our analysis to baricitinib versus placebo comparisons in randomized clinical trials for the treatment of SLE. Observational studies, case studies, conference abstracts, and in vitro and animal experiments were all disregarded.

### Risk of bias assessment

We used the Cochrane risk of bias assessment tool (ROB2) to evaluate the quality of the included articles [21]. ROB2 compromises six domains: the randomized process, deviations from intended interventions, missing outcome data, measurement of outcome, choice of reported findings, and overall risk of bias.

### Data extraction

Using a predetermined data extraction sheet, two different researchers independently extracted the data from the included studies, and any inconsistencies were resolved by a third researcher.

Study ID, study design, NCT, site, length of treatment, inclusion criteria, exclusion criteria, and conclusion were included in the summary data. Age, time since onset of SLE, concomitant medications, baseline Systemic Lupus Erythematosus Disease Activity Index-2000 (SLEDAI-2 K) score, (SLEDAI-2 K) score organ system involvement, Physician’s Global Assessment of Disease Activity (PGA), Cutaneous Lupus Erythematosus Disease Area and Severity Index (CLASI) activity score, tender joint counts (TJC), swollen joint counts (SJC), Systemic Lupus International Collaborating Clinics/ American College of Rheumatology (SLICC/ACR) Damage Index score was among the baseline characteristics.

### Study outcomes

Efficacy outcomes included Systemic Lupus Erythematosus Responder Index-4 (SRI-4) as the primary outcome, reduction of 4 points or more from baseline in Systemic Lupus Erythematosus Disease Activity Index-2000 (SLEDAI-2 K) score, SLEDAI-2 K remission of arthritis or rash, no new British Isles Lupus Assessment Group (BILAG) A and no more than one new (BILAG) B disease activity score, no worsening in Physician Global Assessment (PGA), glucocorticoid sparing, lupus low disease activity state (LLDAS). while safety outcomes included Treatment-emergent adverse events (TEAEs) Discontinuation from study treatment because of an adverse event, infections, serious infections, Opportunistic infections, hepatic disorders, and serious adverse effects.

### Data analysis

#### Meta-analysis

In order to compare the baricitinib group with the placebo group, we used Review Manager version 5.4. To determine the differences in safety and efficacy between baricitinib 2 mg and baricitinib 4 mg, we performed subgroup analysis based on dose. For dichotomous data, the Mantel-Haenszel technique was used to display the risk ratio (RR) and 95% confidence intervals (CI); for continuous data, the inverse variance method was used to display the mean difference (MD) and 95% CI. When P < 0.05, there were significant differences. To assess the degree of heterogeneity, we conducted Chi-Square and I^2^ tests. When the I^2^ > 50% and the P-value of the Chi-square < 0.1, there was significant heterogeneity. If the data were heterogeneous, we would apply a leave-one-out sensitivity analysis or a random effect model. A fixed effect model was utilized in the other cases.

#### Meta-regression

Using Comprehensive meta-analysis (CMA) software, we conducted meta regression analysis to determine if the dose of baricitinib, age of patients, sample size of the study and time since onset of SLE have influenced the primary outcome (SRI-4).

## Results

**Fig. 1:**
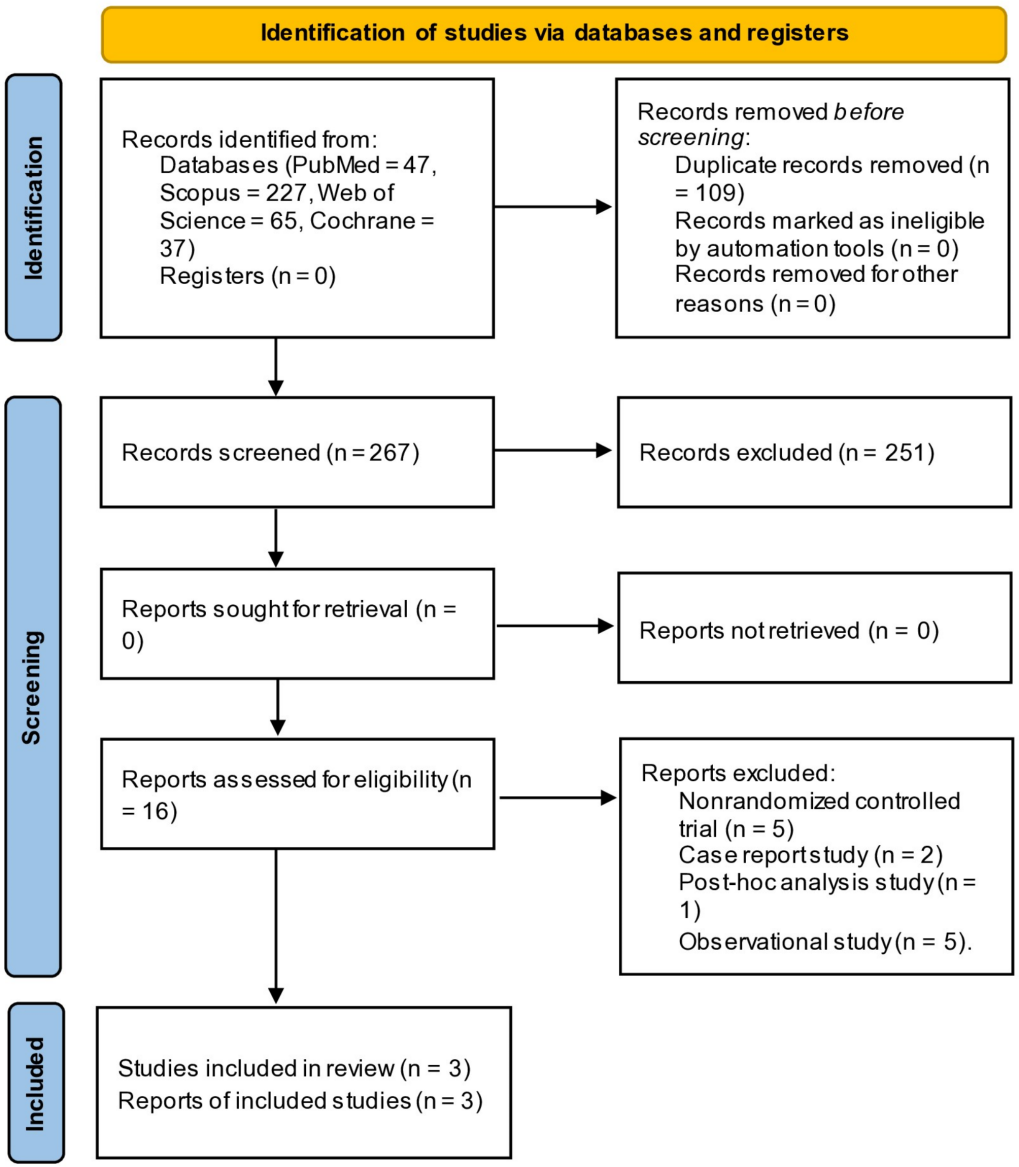
PRISMA flow diagram

After removing duplicates with the systematic review accelerator tool [20], the search turned up a total of 267 citations out of 376 citations. Additionally, two writers looked at the title and abstract of 267 citations and concluded that 16 of them could move on to the full-text screening stage. Finally, three of the 16 citations were included in this meta-analysis [15–17]. All discrepancies were resolved by the third author. PRISMA flow diagram is shown in. (Fig. 1)

In our study, there were 1849 participants, with a mean age of 43.1 years, a mean SLEDAI-2 K score of 9.7, and a prevalence of glucocorticoid use of 77.3%. 621 (33.6%) received 2 mg of baricitinib, 614 (33.2%) received 4 mg of baricitinib, and 614 (33.2%) received a placebo. The summary and baseline characteristics of the included studies are displayed in (Table 1) and (Table 2).

**Table 1:** Summary of included studies

ID	NCT	Study design	Site	Duration of TTT	Inclusion criteria	Exclusion criteria	Conclusion
Petri 2023	NCT03616964	Phase 3	USA, Central America, Mexico, Argentina, Chile, Colombia, France, Italy, Poland, Romania, Spain, Japan, Korea, Philippines, India, Serbia, and South Africa.	52 weeks	Participants required to be at least 18 years old, have a clinical diagnosis of SLE at least 24 weeks before screening, and meet four out of the eleven updated ACR criteria for SLE categorization. They also had a total SLEDAI-2 K score of at least 6 at screening and a clinical SLEDAI-2 K score of at least 4 at baseline, at least one BILAG A score or two BILAG B scores and were positive for at least one of the following: ANA, anti-dsDNA, or anti-Smith.	If a participant had severe active lupus nephritis, active CNS lupus, or another systemic inflammatory disease besides SLE, they were excluded from the study.	Baricitinib was proposed as a potential treatment for SLE patients in phase 2 data, which was supported in SLE-BRAVE-I, however this finding was not repeated in SLE-BRAVE-II. There were no new warning signs of safety.
Morand 2023	NCT03616912	Phase 3	China, Taiwan, Central America, Mexico, Brazil, Austria, Belgium, Croatia, Czech Republic, Germany, Greece, Hungary, Switzerland, Netherlands, UK, USA, Australia, and Russia.	52 weeks	Participants required to be at least 18 years old, have a clinical diagnosis of SLE at least 24 weeks before screening, and meet four out of the eleven updated ACR criteria for SLE categorization. Additionally, they had at least one ANA (titre ≥ 1:80), anti-dsDNA, or anti-Smith positive screening result; active disease indicated by a screening SLEDAI-2 K score of at least 6; and at least one BILAG A score or two BILAG B scores despite SOC treatments.	Patients were disqualified if they had severe active CNS lupus, severe active lupus nephritis, had received treatment for, or were currently experiencing, another systemic inflammatory disease other than SLE.	The 4 mg baricitinib group in this study achieved the primary goal. However, important auxiliary endpoints weren’t. There were no new warning signs of safety.
Wallace 2018	NCT02708095	Phase 2	11 countries in Asia, Europe, North America, and South America.	24 weeks	Participants were to be 18 years of age or older and have received a diagnosis of SLE at least 24 weeks prior to screening by meeting four or more of the 2012 SLICC classification criteria or the new ACR criteria for SLE classification. Patients had to have a positive anti-dsDNA; ≥ 30 IU/mL, a positive ANA test (HEp-2 titre 1:80), a positive SLEDAI-2 K score of 4 or above based on clinical symptoms, or active arthritis or rash as defined by the SLEDAI-2 K. The study medicine was added to already-present stable background SOC, which may have included corticosteroids or nonsteroidal anti-inflammatory drugs.	Active severe lupus nephritis, active severe CNS lupus, a recent clinically acute infection, and a few specific test abnormalities were important exclusion criteria.	With a safety profile consistent with prior baricitinib studies, the 4 mg dose of baricitinib, but not the 2 mg dose, significantly improved the signs and symptoms of active SLE in patients who were not sufficiently managed despite SOC. This research lays the groundwork for upcoming phase 3 trials using the possible oral treatment for SLE, baricitinib.

**Abbreviations**: SLE, systemic lupus erythematosus; ACR, American college of rheumatology; SLEDAI-2 K, systemic Lupus Erythematosus Disease Activity Index 2000; BILAG, british isles lupus assessment group; ANA, anti-nuclear antibody; anti-dsDNA, anti-double stranded DNA; CNS, central nervous system; SOC, standard of care

**Table 2:** Baseline characteristics of included participants

ID	Duration	Dose	Age (years), M (SD)	Time since onset of SLE, M (SD)	Concomitant medications							
					**Glucocorticoids, N (%)**	**Prednisone dose (mg/day), M (SD)**	**Antimalarials, N (%)**	**Immunosuppressants, N (%)**	**Methotrexate, N (%)**	**Azathioprine, N (%)**	**Mycophenolate mofetil, N (%)**	**NSAIDs, N (%)**
**Petri 2023**	**52 weeks**	2 mg	42·8 (13·0)	8·7 (7·7)	210 (80%)	9·6 (5·9)	213 (82%)	133 (51%)	52 (20%)	48 (18%)	25 (10%)	64 (25%)
		4 mg	42·2 (12·1)	8·5 (7·7)	207 (80%)	9·8 (5·7)	213 (83%)	133 (52%)	60 (23%)	33 (13%)	25 (10%)	70 (27%)
		Placebo	43·5 (13·5)	9·0 (8·3)	207 (81%)	8·8 (5·0)	209 (82%	140 (55%)	49 (19%)	47 (18%)	29 (11%)	51 (20%)
**Morand 2023**	**52 weeks**	2 mg	42·9 (12·4)	9·2 (7·7)	194 (76%)	10·4 (7)	189 (74%)	152 (60%)	51 (20%)	54 (21%)	39 (15%)	67 (26%)
		4 mg	41·5 (12·9)	8·8 (8·2)	187 (74%)	10·1 (6)	206 (82%)	141 (56%)	52 (21%)	42 (17%)	34 (14%)	68 (27%)
		Placebo	42·0 (12·0)	9·4 (7·5)	195 (77%)	9·8 (5)	213 (84%)	150 (59%)	60 (24%)	38 (15%)	39 (15%)	63 (25%)
**Wallace 2018**	**24 weeks**	2 mg	43·2 (11·0)	11·8 (9·1)	79 (75%)	8·7 (5·8)	71 (68%)	47 (45%)	17 (16%)	10 (10%)	10 (10%)	29 (28%)
		4 mg	45·0 (12·4)	11·5 (10·3)	74 (71%)	10·5 (17·4)	76 (73%)	50 (48%)	13 (13%)	11 (11%)	16 (15%)	32 (31%)
		Placebo	44·9 (12·8)	9·7 (7·7)	77 (73%)	7·9 (4·6)	75 (71%)	45 (43%)	13 (12%)	15 (14%)	11 (10%)	27 (26%)
**SLEDAI-2 K score, M (SD)**	**SLEDAI-2 K score ≥ 10, N (%)**	**SLEDAI-2 K organ system involvement**									**PGA score, M (SD)**	**CLASI activity score, M (SD)**
		**CNS, N (%)**	**Vascular, N (%)**	**Musculoskeletal, N (%)**	**Renal, N (%)**	**Mucocutaneous, N (%)**	**Cardiovascular and respiratory, N (%)**	**Immunological, N (%)**	**Constitutional, N (%)**	**Hematological, N (%)**		
10·1 (3·4)	149 (57%)	0	11 (4%)	253 (97%)	22 (8%)	252 (97%)	6 (2%)	143 (55%)	10 (4%)	19 (7%)	61·0 (12·5)	6·6 (6·6)
10·1 (3·0)	144 (56%)	0	14 (5%)	251 (97%)	10 (4%)	251 (97%)	9 (3%)	132 (51%)	11 (4%)	17 (7%)	58·8 (14·6)	6·7 (5·8)
10·1 (3·2)	147 (57%)	0	12 (5%)	252 (98%)	18 (7%)	243 (95%)	4 (2%)	138 (54%	8 (3%)	20 (8%)	60·0 (14·6)	6·8 (6·1)
10·3 (3)	152 (60%)	0	15 (6%)	250 (98%)	24 (9%)	246 (97%)	6 (2%)	131 (51%)	3 (1%)	13 (5%)	1·8 (0·4)	6·5 (7·0)
10·0 (3)	146 (58%)	0	10 (4%)	247 (98%)	17 (7%)	241 (96%)	8 (3%)	137 (54%)	2 (1%)	21 (8%)	1·8 (0·5)	6·1 (5·6)
10·1 (3)	144 (57%)	0	15 (6%)	247 (98%)	15 (6%)	244 (96%)	10 (4%)	134 (53%)	4 (2%)	13 (5%)	1·8 (0·5)	6·3 (6·2)
8·8 (3·4)	35 (33%)	1 (1%)	4 (4%)	93 (89%)	9 (9%)	82 (78%)	1 (1%)	63 (60%)	2 (2%)	9 (9%)	48·8 (15·8)	3·8 (5·4)
9·0 (3·3)	44 (42%	2 (2%)	3 (3%)	96 (92%)	7 (7%)	92 (88%)	1 (1%)	64 (62%)	0	5 (5%)	51·7 (16·0)	4·0 (3·4)
8·9 (2·9)	43 (41%)	3 (3%)	1 (1%)	93 (89%)	9 (9%)	90 (86%)	2 (2%)	62 (59%)	2 (2%)	13 (12%)	49·5 (16·9)	4·9 (5·7)
**TJC, M (SD)**	**SJC, M (SD)**	**SLICC/ACR Damage Index score, m (SD)**										
10·3 (6·8)	6·64 (5·1)	0·68 (1·1)										
10·6 (7·1)	6·68 (5·0)	0·60 (1·0)										
10·6 (7·1)	6·41 (5·4)	0·66 (1·1)										
10·3 (6·5)	6·9 (5·1)	0·6 (1·0)										
10·9 (7·0)	6·9 (5·4)	0·6 (1·0)										
10·0 (7·0)	7·1 (5·6)	0·6 (1·0)										
8·7 (6·6)	5·2 (4·7)	0·44 (0·68)										
8·5 (6·2)	5·5 (4·2)	0·40 (0·88)										
7·7 (5·8)	5·3 (4·7)	0·59 (0·97)										

**Abbreviations**: M, mean; SD, standard deviation; SLE, systemic lupus erythematosus; N, numbers; SLEDAI-2 K, systemic Lupus Erythematosus Disease Activity Index 2000; CNS, central nervous system; PGA; physician global assessment; CLASI, Cutaneous Lupus Erythematosus Disease Area and Severity Index; TJC, tender joint count; SJC, swollen joint count; SLICC/ACR, Systemic International Collaborating Clinics/American College of Lupus Rheumatology

### Risk of bias results

**Fig. 2:**
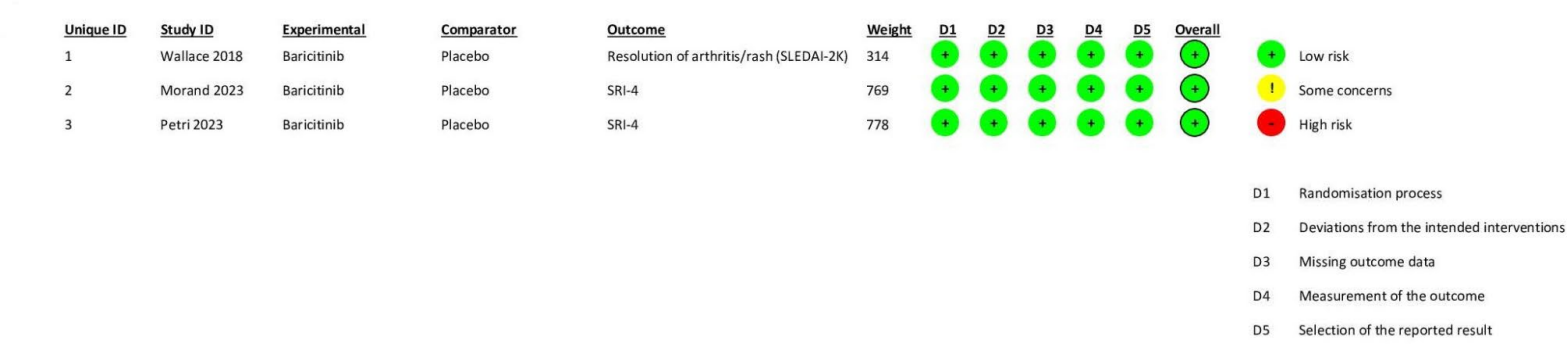
Risk of bias graph

All of the included studies had a low risk of bias. (Fig. 2) and (S1) respectively exhibit a graph and summary of the bias risk.

### Meta-analysis

#### Efficacy outcomes

##### SRI-4

**Fig. 3:**
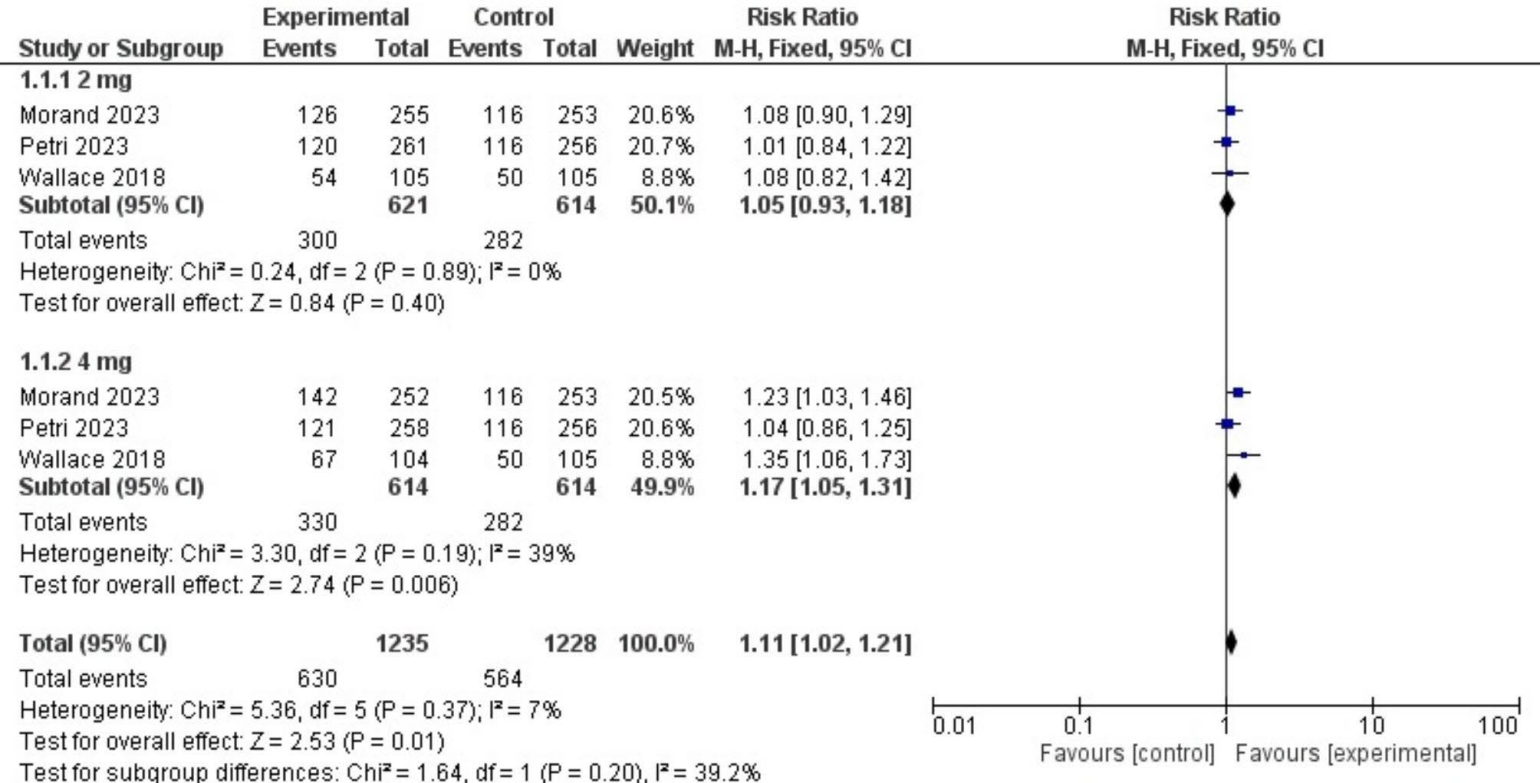
A forest plot displaying the prevalence of SLE Responder Index-4 in the baricitinib group versus the placebo group

Participants in the baricitinib group were significantly more likely to obtain an SRI-4 response than those in the placebo group [RR = 1.13, 95% CI (1.04, 1.22), P = 0.004]. Moreover, baricitinib 4 mg significantly outperformed placebo in the sub-group analysis [RR = 1.18, 95% CI (1.06, 1.32), P = 0.003]. (Fig. 3)

##### Reduction of ≥ 4 points from baseline in SLEDAI-2 K score

**Fig. 4:**
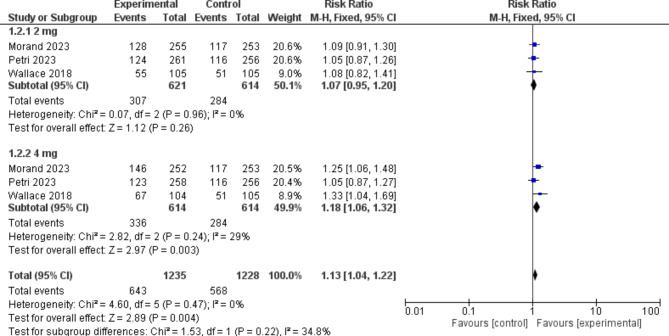
A forest plot displaying the prevalence of Reduction of ≥ 4 points from baseline in Systemic Lupus Erythematosus Disease Activity Index 2000 score in the baricitinib group versus the placebo group

Participants in the baricitinib group were significantly more likely to obtain a reduction of *≥ 4 points from baseline in SLEDAI-2 K score* than those in the placebo group [RR = 1.13, 95% CI (1.04, 1.22), P = 0.004]. Moreover, baricitinib 4 mg significantly outperformed the placebo in the sub-group analysis [RR = 1.18, 95% CI (1.06, 1.32), P = 0.003]. (Fig. 4)

##### SLEDAI-2 K remission of arthritis or rash

**Fig. 5:**
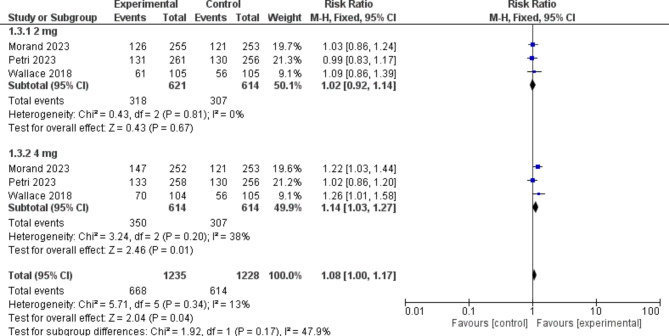
A forest plot displaying the prevalence of Systemic Lupus Erythematosus Disease Activity Index 2000 remission of arthritis or rash in the baricitinib group versus the placebo group

Participants in the baricitinib group were significantly more likely to obtain SLEDAI-2 K remission of arthritis or rash than those in the placebo group [RR = 1.08, 95% CI (1.00, 1.17), P = 0.04]. Moreover, baricitinib 4 mg significantly outperformed placebo in the sub-group analysis [RR = 1.14, 95% CI (1.03, 1.27), P = 0.01]. (Fig. 5)

##### No new BILAG A and no more than one new BILAG B disease activity score

**Fig. 6:**
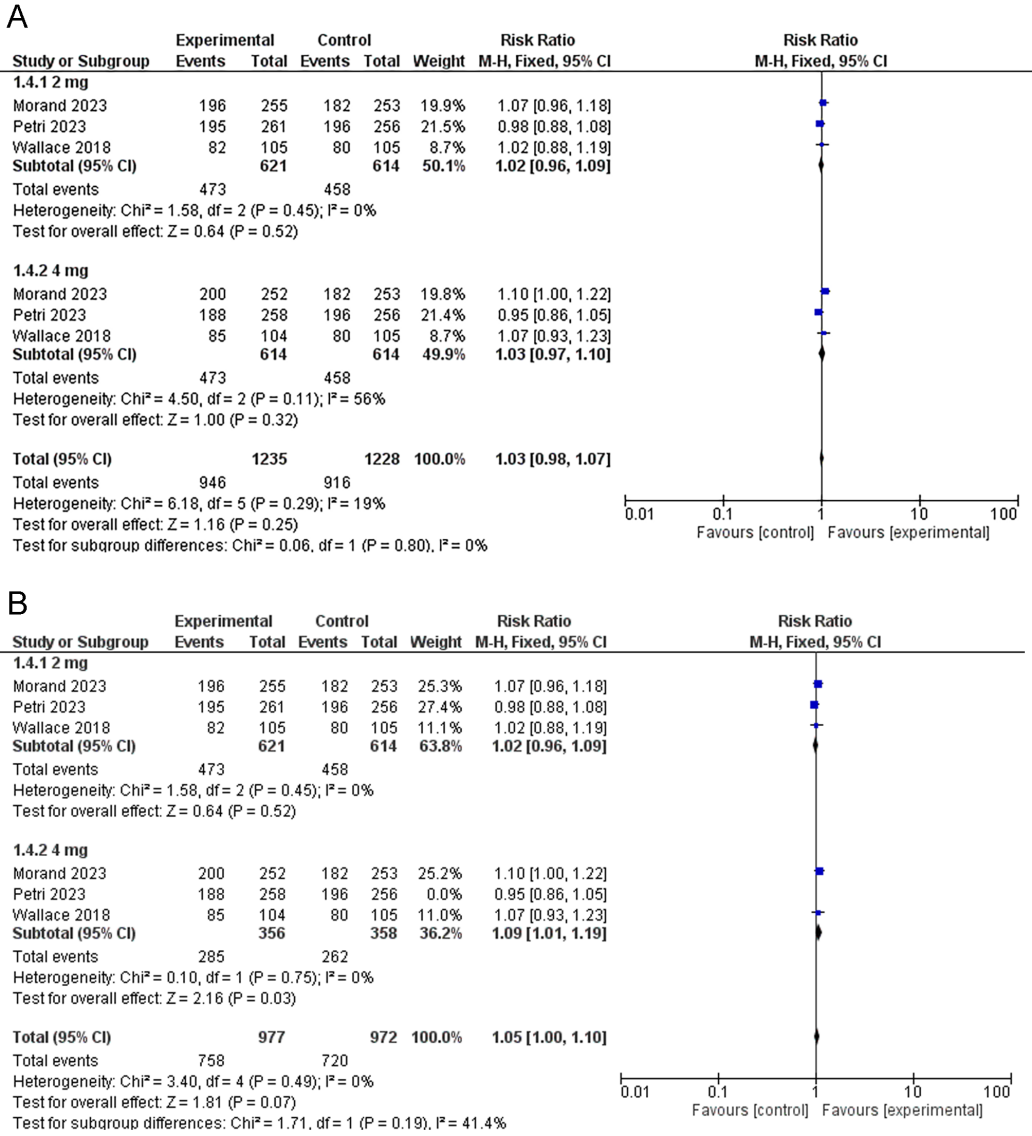
A forest plot displaying the prevalence of No new BILAG A and no more than one new BILAB B disease activity score in the baricitinib group versus the placebo group. (**A**) before sensitivity analysis. (**B**) after sensitivity analysis

In terms of BILAG, there was no significant difference between the baricitinib group and the placebo group [RR = 1.03, 95% CI (0.98, 1.07), P = 0.25]. Similarly, there was no significant difference between baricitinib 4 mg and placebo in the sub-group analysis [RR = 1.03, 95% CI (0.97, 1.10), P = 0.32]. The data, however, were heterogeneous [P = 0.11, I^2^ = 56%]. I took out Petri et al. [17] to address this heterogeneity. The outcome was altered and baricitinib 4 mg was significantly better than the placebo [RR = 1.09, 95% CI (1.01, 1.19), P = 0.03]. (Fig. 6A&B)

##### No worsening by PGA

**Fig. 7:**
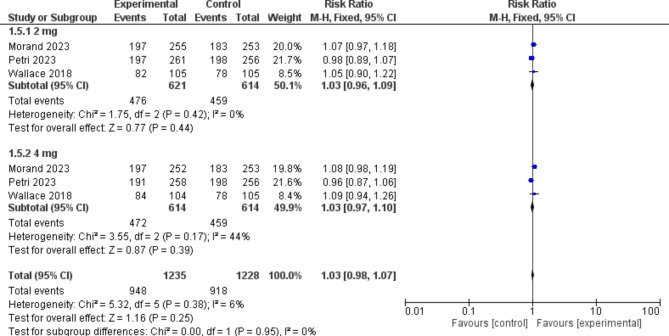
A forest plot displaying the prevalence of No worsening by PGA in the baricitinib group versus the placebo group

In terms of PGA, there was no significant difference between the baricitinib group and the placebo group [RR = 1.03, 95% CI (0.98, 1.07), P = 0.25]. (Fig. 7)

##### Glucocorticoid sparing

The glucocorticoid-sparing outcome was reported in two studies [16, 17]. There was no significant difference between the baricitinib group and the placebo group [RR = 1.02, 95% CI (0.84, 1.24), P = 0.86]. (Figure S2)

##### LLDAS

In terms of LLDAS, there was no significant difference between the baricitinib group and the placebo group [RR = 1.12, 95% CI (0.98, 1.27), P = 0.1]. (Figure S3)

##### Participants with ≥ 1 severe flare

In terms of participants with ≥ 1 severe flare, there was no significant difference between the baricitinib group and the placebo group [RR = 0.88, 95% CI (0.70, 1.09), P = 0.24]. (Figure S4)

##### SLEDAI-2 K “score”

The baricitinib group achieved a lower SLEDAI-2 K score than the placebo group, indicating a significant difference between the two groups [MD = -0.44, 95% CI (-0.72, -0.15), P = 0.003]. Moreover, baricitinib 4 mg significantly outperformed the placebo in the sub-group analysis [MD = -0.67, 95% CI (-1.08, -0.26), P = 0.001]. (Figure S5)

#### Safety outcomes

##### TEAEs

**Fig. 8:**
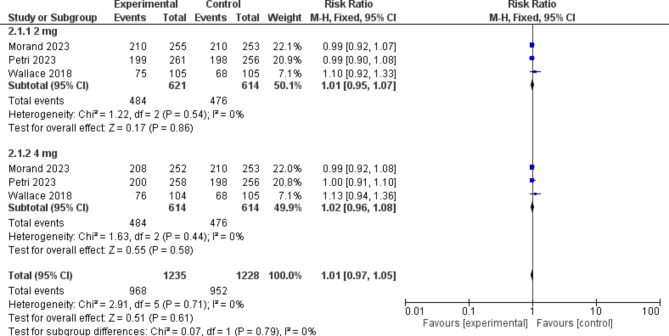
A forest plot displaying the prevalence of treatment emergent adverse events in the baricitinib group versus the placebo group

The baricitinib group and the placebo group did not differ significantly in terms of TEAEs [RR = 1.01, 95% CI (0.97, 1.05), P = 0.61]. Moreover, there was no significant difference between the baricitinib group and the placebo group in terms of mild, moderate, or severe TEAEs. (Fig. 8)

##### Serious adverse events

Participants in the baricitinib group were significantly more likely to obtain serious AEs than those in the placebo group [RR = 1.49, 95% CI (1.16, 1.92), P = 0.002]. (Figure S6)

##### Discontinuation from study because of an adverse event

Regarding Discontinuation from study treatment because of an AE, there was no significant difference between the baricitinib group and the placebo group [RR = 1.19, 95% CI (0.92, 1.54), P = 0.19]. (Figure S7)

##### Infections and serious Infections

The baricitinib group and the placebo group did not differ significantly in terms of infections [RR = 1.03, 95% CI (0.95, 1.12), P = 0.46]. However, participants in the baricitinib group were significantly more likely to obtain serious infections than those in the placebo group [RR = 2.07, 95% CI (1.28, 3.34), P = 0.003]. (Figures S8 and S9)

### Opportunistic infection

Participants in the baricitinib group were significantly more likely to obtain opportunistic infection than those in the placebo group [RR = 1.47, 95% CI (1.02, 2.11), P = 0.04]. (Figure S10)

#### Hepatic disorders

The baricitinib group and the placebo group did not differ significantly in terms of hepatic disorders. (Figure S11)

#### Meta-regression

Random effect meta-regression was applied for dose of baricitinib, age, sample size of the study and time since onset of SLE regarding SRI-4. There was no significant association between SRI-4 outcome and age of patients [OR = 0.0104, 95% CI (-0.2488, 0.2697), P = 0.937, figure S12], sample size [OR = -0.0005, 95% CI (-0.0024, 0.0014), P = 0.6093, figure S13], dose of baricitinib [OR = 0.0371, 95% CI (-0.2238, 0.2979), P = 0.7805, figure S14], or time since onset of SLE[OR = 0.1441, 95% CI (-0.1594, 0.4476), P = 0.352, figure S15].

## Discussion

Baricitinib has emerged as a potential therapeutic option for the treatment of wide scale autoimmune conditions including SLE due to its ability to modulate multiple cytokines involved in the pathogenesis of the disease [14]. Recent clinical trials have evaluated the safety and efficacy of baricitinib in SLE [15–17]. Herrin, we performed a meta-analysis utilizing data from these trials. Based on the literature search, we believe that our study is the first meta-analysis to evaluate the safety and efficacy of baricitinib in the treatment of SLE.

In terms of efficacy, the overall analysis demonstrated that baricitinib achieved the primary endpoint of a higher proportion of patients reaching an SRI-4 response compared with placebo. Our results were in line with the results of phase three RCT reported by Morand et al. [16]. In contrast, Petri et al. reported no significant SRI-4 response in baricitinib group compared with placebo [17]. The profile of patients, including organ involvement, in both trials was almost similar suggesting that patient clinical and immunological characteristics were not a major factor. Furthermore, the results of meta-regression analysis demonstrated no significant effect of age of patients, sample size, baricitinib dose, or time since onset on the primary outcome (SRI-4).

Furthermore, baricitinib demonstrated superiority over placebo in terms of BILAG A or B disease activity score. Additionally, it outperformed placebo in SLEDAI-2 K score of 4 points or more from baseline as well as a decrease in rash or arthritis based on the SLEDAI-2 K score. This adds a potential impact on reducing specific disease symptoms and activity. Our observations on these outcomes align with the results obtained from preclinical models. Preclinical studies conducted on (MRL/lpr) mice and in vitro using immortalized primary podocytes and B cells isolated from C57BL/6 mice have demonstrated significant reductions in disease activity with baricitinib [22].

In terms of safety, Baricitinib appeared to be a well-tolerated drug for SLE. However, it is noteworthy that baricitinib had a higher incidence of serious adverse events and infections. This was similar to the results of pooled analysis on the safety of baricitinib in the treatment of SLE retorted by Dörner T et al. [23].

The results of our study should be interpreted cautiously because we were limited by the small number of clinical trials included. Only three randomized clinical trials were identified, which may have resulted in inaccurate precision of the results. Further research on the effectiveness of the baricitinib drug on patients with SLE is recommended. Moreover, long-term studies are also needed to assess the safety and efficacy of the drug over extended periods. Additionally, investigating the potential benefits of combining baricitinib with other treatments for SLE could provide valuable insights into the optimal management of this complex disease.

## Conclusion

There is now sufficient clinical evidence to support Baricitinib’s safety and efficacy in the treatment of SLE. This study’s primary aim and some secondary endpoints were significantly reached by baricitinib. While glucocorticoids sparing and some other of the secondary outcomes didn’t reach statistical significance, the overall analysis demonstrates a promising efficacy of baricitinib in the management of SLE.

### Electronic supplementary material

Below is the link to the electronic supplementary material.

Supplementary Material 1

## Data Availability

The datasets generated during and/or analyzed during the current study are available from the corresponding author upon reasonable request.

## List of abbreviations

BILAG: British Isles Lupus Assessment Group
LLDAS: Lupus low disease activity state
PGA: Physician Global Assessment
ROB: Risk of Bias Assessment tool
SLEDAI-2K: Systemic Lupus Erythematosus Disease Activity Index-2000)
SRI-4: Systemic Lupus Erythematosus Responder Index-4
TEAEs: Treatment-Emergent Adverse Events
