# Supplementary material for: Efficacy and safety of baricitinib in treatment of systemic lupus erythematosus: a systematic review and meta-analysis
Source: BMC Rheumatol. 2023 Oct 31;7:40. doi: 10.1186/s41927-023-00363-6 (PMC10617176; doi:10.1186/s41927-023-00363-6)
Supplement: Supplementary file 1 — Supplementary Material 1 [file 41927_2023_363_MOESM1_ESM.docx]

**Supplmentary**

**Search strategy**

(“Systemic Lupus Erythematosus” OR “Lupus Erythematosus Disseminatus” OR “Libman-Sacks Disease” OR “Disease, Libman-Sacks” OR “Libman Sacks Disease”) AND (baricitinib OR INCB-28050 OR Olumiant OR “baricitinib phosphate” OR “baricitinib phosphate salt” OR INCB028050 OR INCB-028050 OR LY3009104 OR LY-3009104)

**Supplementary figure legends**

Figure S1: Risk of bias summary.

Figure S2: A forest plot displaying the prevalence of glucocorticoid sparing in the baricitinib group versus the placebo group.

Figure S3: A forest plot displaying the prevalence of Participants with ≥ 1 severe flare in the baricitinib group versus the placebo group.

Figure S4: A forest plot displaying the prevalence of lupus low disease activity state in the baricitinib group versus the placebo group.

Figure S5: A forest plot showing the difference in Systemic Lupus Erythematosus Disease Activity Index 2000 score between the baricitinib group and the placebo group.

Figure S6: A forest plot displaying the prevalence of serious adverse events in the baricitinib group versus the placebo group.

Figure S7: A forest plot displaying the prevalence of Discontinuation from study treatment because of an adverse event in the baricitinib group versus the placebo group.

Figure S8: A forest plot displaying the prevalence of infections in the baricitinib group versus the placebo group.

Figure S9: A forest plot displaying the prevalence of serious infections in the baricitinib group versus the placebo group.

Figure S10: A forest plot displaying the prevalence of opportunistic infections in the baricitinib group versus the placebo group.

Figure S11: A forest plot displaying the prevalence of hepatic disorders in the baricitinib group versus the placebo group.

Figure S12**:** Meta-regression analysis of a log odds ratio of SRI-4 and age of the patients.

Figure S13: Meta-regression analysis of a log odds ratio of SRI-4 and sample size**.**

Figure S14: Meta-regression analysis of a log odds ratio of SRI-4 and dose of baricitinib.

Figure S15: Meta-regression analysis of a log odds ratio of SRI-4 and time since onset of SLE**.**


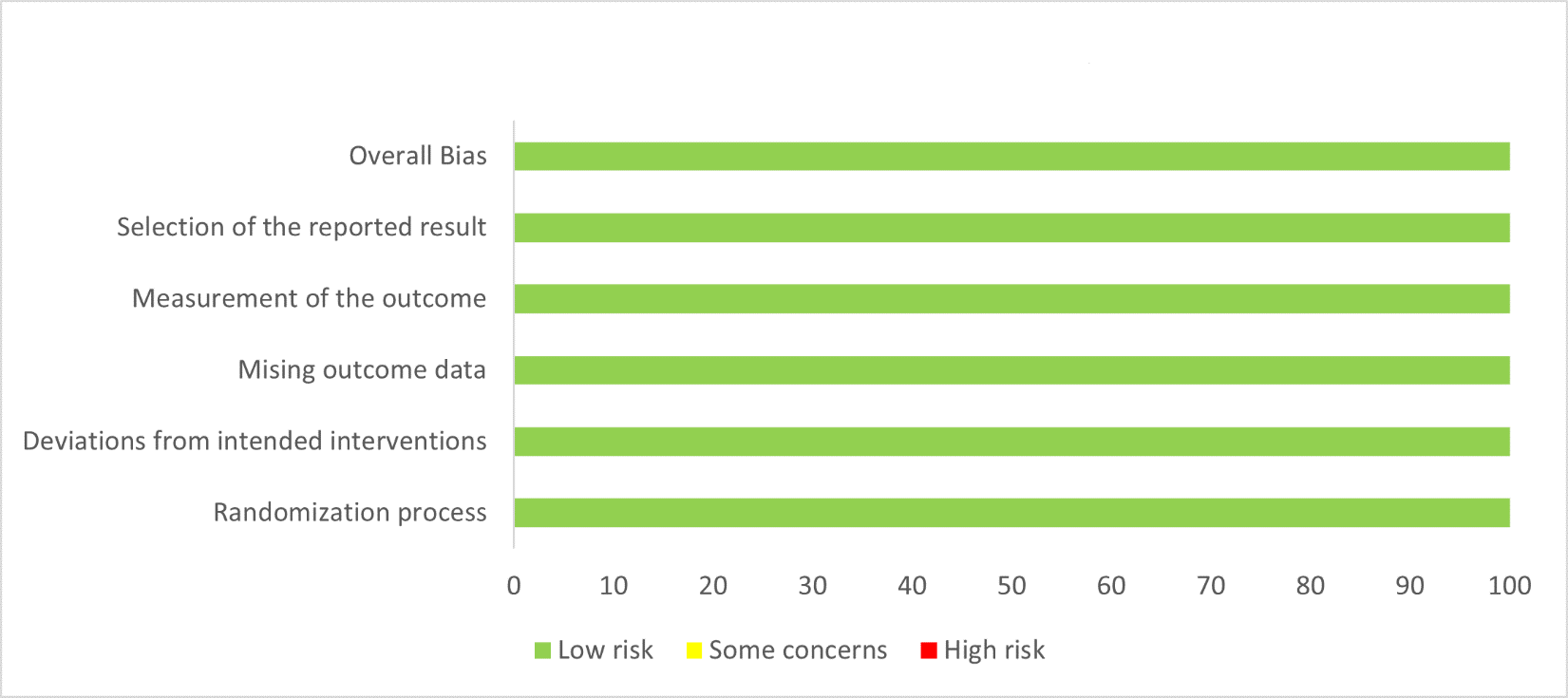


Figure S1: Risk of bias summary.





Figure S2: A forest plot displaying the prevalence of glucocorticoid sparing in the baricitinib group versus the placebo group.





Figure S3: A forest plot displaying the prevalence of lupus low disease activity state in the baricitinib group versus the placebo group.





Figure S4: A forest plot displaying the prevalence of Participants with ≥ 1 severe flare in the baricitinib group versus the placebo group.





Figure S5: A forest plot showing the difference in Systemic Lupus Erythematosus Disease Activity Index 2000 between the baricitinib group and the placebo group.





Figure S6: A forest plot displaying the prevalence of serious adverse events in the baricitinib group versus the placebo group.





Figure S7: A forest plot displaying the prevalence of Discontinuation from study treatment because of an adverse event in the baricitinib group versus the placebo group.





Figure S8: A forest plot displaying the prevalence of infections in the baricitinib group versus the placebo group.





Figure S9: A forest plot displaying the prevalence of serious infections in the baricitinib group versus the placebo group.





Figure S10: A forest plot displaying the prevalence of opportunistic infections in the baricitinib group versus the placebo group.





Figure S11: A forest plot displaying the prevalence of hepatic disorders in the baricitinib group versus the placebo group.

Figure S12**:** Meta-regression analysis of a log odds ratio of SRI-4 and age of the patients


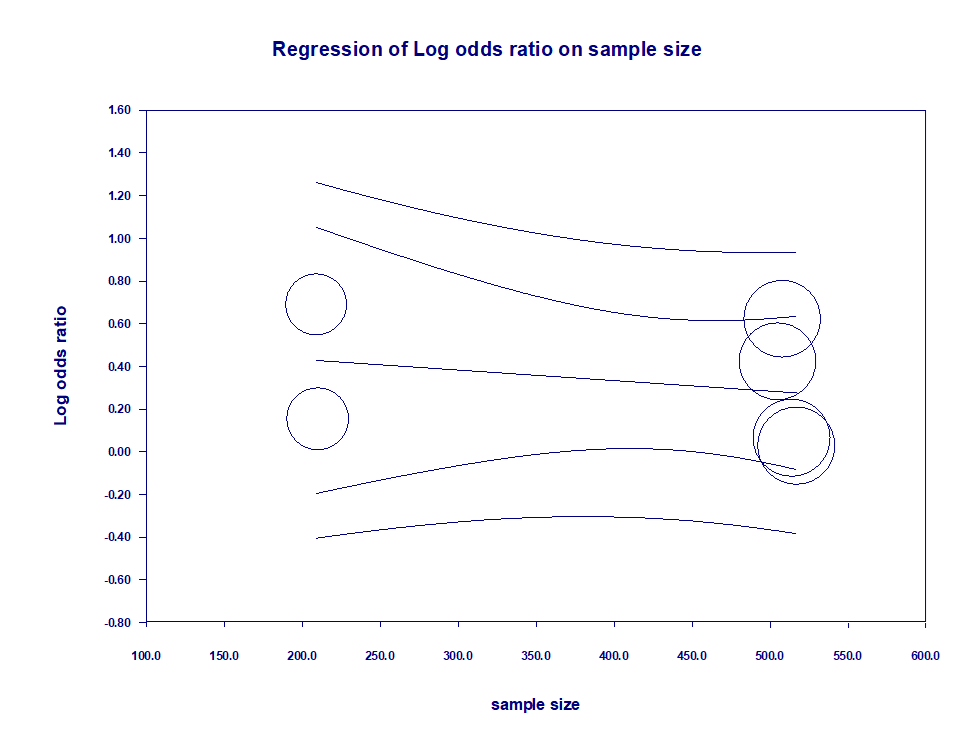


Figure S13: Meta-regression analysis of a log odds ratio of SRI-4 and sample size


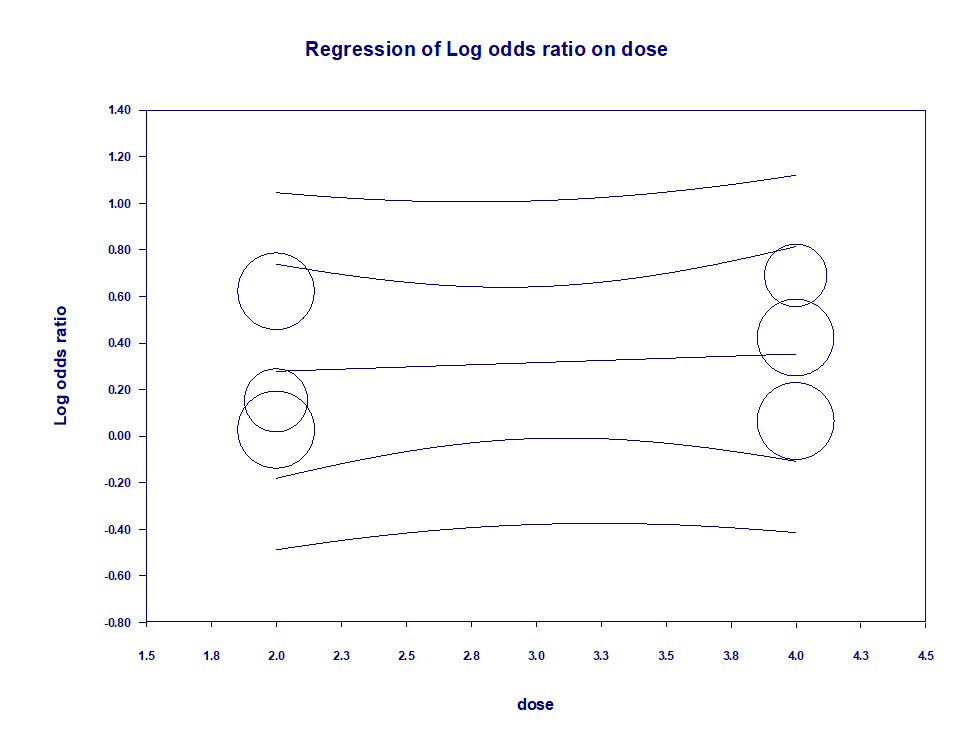


Figure S14: Meta-regression analysis of a log odds ratio of SRI-4 and dose of baricitinib


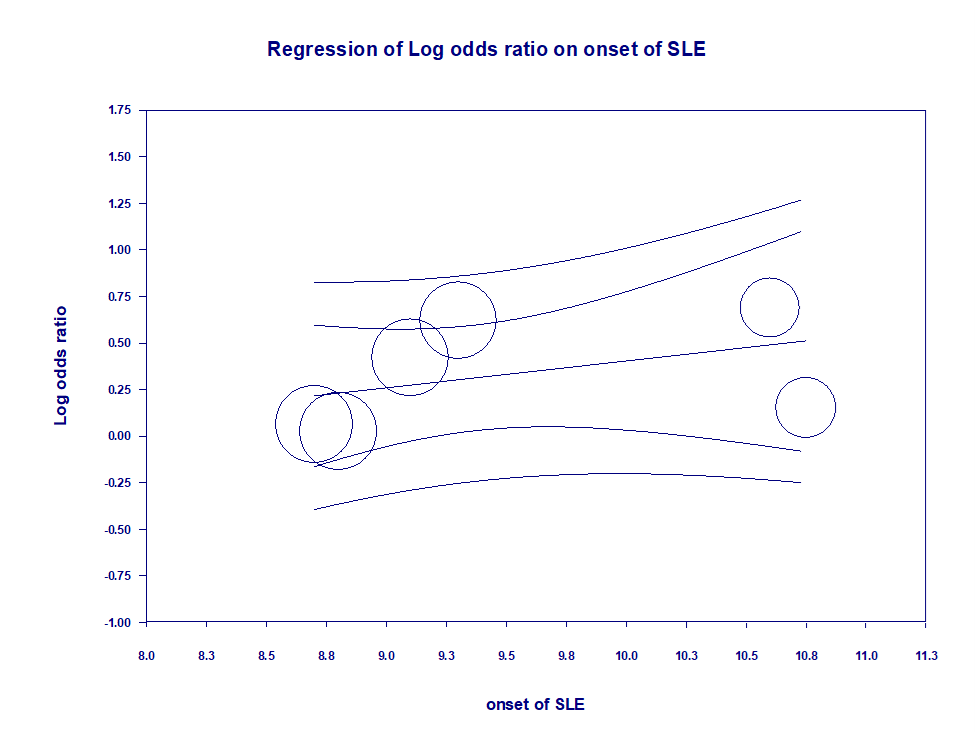


Figure S15: Meta-regression analysis of a log odds ratio of SRI-4 and time since onset of SLE
